# Transcriptomic adaptation of skeletal muscle in response to MICT and HIIT exercise modalities

**DOI:** 10.1371/journal.pone.0318782

**Published:** 2025-02-25

**Authors:** Weihao Hong, Yisheng Luan, Jianrong Zheng, Yingzhe Xiong, Bing Zhang, Yixuan Ma

**Affiliations:** 1 Division of Sports Science and Physical Education, Tsinghua University, Beijing, China; 2 Department of Prevention and Early Warning Research, Guangzhou National Laboratory, Guangzhou, China; 3 School of Physical Education, Central China Normal University, Wuhan, China; Erzurum Technical University: Erzurum Teknik Universitesi, TÜRKIYE

## Abstract

Skeletal muscle exhibits remarkable plasticity in response to diverse stimuli, with exercise serving as a potent trigger. Varied exercise modalities, including moderate-intensity continuous training (MICT) and high-intensity interval training (HIIT), induce distinct structural and functional adaptations on skeletal muscle. However, the underlying molecular mechanisms governing these adaptations remain poorly understood. In this study, we utilized RNA-seq to characterize the transcriptomic profile of murine gastrocnemius muscle following 8-week treadmill-based MICT (M group) and HIIT (H group). A total of 1052 DEGs were screened in H vs. M. Among the top 10 significant DEGs, *Foxo1* and *Myod1* are closely related to muscular physiology. Through KEGG pathway analysis, distinct adaptations were primarily identified in the FoxO, MAPK, and PI3K-AKT pathways. By analyzing the expression of myokines, a significantly higher *Igf-1* expression level was observed in the M group compared to the H group. Therefore, IGF-1, a well-known upstream regulator of both the PI3K-AKT-FoxO and MAPK pathways, might drive distinct muscle adaptations through variations in *Igf-1* expression induced by these two exercise modalities.

## Introduction

Skeletal muscle is a dynamic and versatile tissue capable of adapting to functional demands imposed by a myriad of internal and external stimuli [1]. Among these stimuli, exercise serves as a potent trigger, inducing a cascade of physiological changes within skeletal muscle [2–4]. Such changes encompass alterations in contractile machinery, structural organization, and metabolic pathways within muscle fibers. Additionally, exercise enhances capillary density and promotes the remodeling of connective tissue. Together, these processes underpin skeletal muscle adaptations that are closely linked to the specific type of exercise undertaken [5,6].

Among the various exercise modalities, moderate-intensity continuous training (MICT) and high-intensity interval training (HIIT) are two exercise modalities with distinct characteristics and benefits [7]. MICT involves moderate-intensity, long-duration aerobic activities such as jogging, cycling, or swimming, typically lasting over 30 minutes. This approach is favored for its safety, stability, and ease of adherence, rendering it effective in improving cardiovascular endurance, fat loss, and overall stamina [8,9]. In contrast, HIIT consists of alternating brief bursts of high-intensity exercise, such as sprints, with short recovery periods [10]. It provides fitness benefits within a shorter time frame, particularly in enhancing metabolism, fat loss, muscle strength, and cardiovascular function [11,12].

Although previous studies on MICT and HIIT have provided valuable insights into their distinct effects on physiological and functional outcomes [13,14], the molecular mechanisms governing skeletal muscle adaptation to these exercise modalities remain poorly explored. Bridging this gap is critical for understanding how different types of exercise shape muscle plasticity at the molecular level, paving the way for optimized training protocols and personalized exercise strategies. In particular, the growing recognition of exercise as a therapeutic intervention for a wide range of health conditions underscores the urgency of unraveling the transcriptional basis of muscle adaptation to different exercise types.

This study employs RNA-seq to characterize the transcriptomic profile of murine gastrocnemius muscle following an 8-week treadmill-based MICT/HIIT regimen. By elucidating the molecular pathways underpinning the distinct adaptations to these two modalities, this study contributes to a deeper understanding of skeletal muscle plasticity and provides valuable insights for sports science and therapeutic strategies.

## Materials and methods

### Animals

14-week-old male C57BL/6J mice were housed in SPF-grade rooms, with controlled temperature (22°C ± 2°C) and humidity (50% ± 5%). Mice were divided into three groups (each n = 12): sedentary control (C), MICT (M), and HIIT (H). All mice were purchased from Tsinghua Laboratory Resource Center. This study was approved by the Institutional Animal Care and Use Committee of Tsinghua University (Approval No: THU-LARC-2023-004) and was conducted in accordance with the ARRIVE guidelines. All animal methods were carried out in accordance with relevant guidelines and regulations.

### Exercise protocol

MICT: Adaptation training was conducted for 1 week at a speed of 10 m/min for 15 minutes per day. Formal training was conducted for 8 weeks, with sessions lasting 45 minutes per day, five days a week. To monitor exercise intensity, a maximal oxygen uptake (VO_2_max) test was conducted every two weeks using the TSE Systems Phenomaster (S1 Table). The MICT training regimen: 15 m/min for weeks 1-2, 17 m/min for weeks 3-4, and 19 m/min for weeks 5-8.

HIIT: Adaptation training was conducted for 1 week at a speed of 10 m/min for 15 minutes per day. Formal training was conducted for 8 weeks, five days a week. Each HIIT session consisted of 1 minute of high-intensity exercise (90% of VO_2_max, 24 m/min for weeks 1-2, 26 m/min for weeks 3-4, 28 m/min for weeks 5-8) followed by 2 minutes of moderate-intensity exercise (60% of VO_2_max, same as MICT). The number of HIIT repetitions was adjusted to match the total distance covered by the MICT group.

### Body weight and serum parameter measurements

Body weight was measured and recorded weekly. Following intraperitoneal injection of Avertin for anesthesia, blood was drawn from the eye. Serum was separated by centrifugation at 3000 rpm for 15 minutes at 4 °C, and serum triglyceride and glucose levels were quantified using an automated chemistry analyzer (Kehua ZY KHB1280).

### Gastrocnemius muscle RNA-seq

RNA-seq was conducted at Biomarker (Beijing, China). Paired-end reads were quality checked and trimmed using Trim-galore (v.0.6.0). Alignment of the reads to the mouse genome reference (GRCm38.p6) from GENCODE was performed using STAR (v.2.7.3a). Subsequently, featureCounts (v.1.6.3) was utilized to count reads mapped to exon sites of genes listed in GTF files obtained from GENCODE. Differential expression analysis was carried out using DESeq2 (v.1.22.2), with raw counts as input. Functional enrichment analyses, including KEGG and GO analyses of differentially expressed genes (DEGs), were performed using clusterProfiler (v3.10.1).

### Quantitative real-time PCR

Gastrocnemius muscle (each group n = 6) total RNA extraction was performed with Trizol (Invitrogen) according to the manufacturer’s instructions. cDNA was synthesized using HiScript II Q RT SuperMix (Vazyme). Quantitative RT-PCR was performed using AceQ qPCR SYBR Green Master Mix (Vazyme). The experiment was independently repeated three times.

The following primers were used:

*Gapdh* Forward: AGAAGGTGGTGAAGCAGGCATCT
  Reverse: CGGCATCGAAGGTGGAAGAGTG
*Igf-1* Forward: CTGGACCAGAGACCCTTTGC
  Reverse: GGACGGGGACTTCTGAGTCTT
*Sparc* Forward: GTGGAAATGGGAGAATTTGAGGA
  Reverse: CTCACACACCTTGCCATGTTT

### Statistical analysis

GraphPad Prism 7 (Graphpad software) was used to assess statistical significance. The data are presented as x̄ ± s. One-way analysis of variance (ANOVA) was used for comparisons between multiple groups followed by Tukey’s post-hoc test, *p* < 0.05 was considered statistically significant.

## Results

### Physiological parameters

To investigate the transcriptional adaptation of skeletal muscle in response to MICT and HIIT, 14-week-old C57BL/6J mice were divided into three groups: sedentary control (C), MICT (M), HIIT (H). After grouping, the mice were initially weighed (referred to as week 1) and then underwent an 8-week treadmill MICT/HIIT regimen following a one-week adaptative training. As shown in Table 1, the body weight of mice in each group remained relatively stable throughout the experimental period. Additionally, no significant differences were observed in blood glucose, blood triglyceride, gastrocnemius muscle mass and gastrocnemius muscle index (the ratio of bilateral gastrocnemius muscle weight to body weight) among the groups (Table 2).

**Table 1:** Body weight.

Week	C	M	H
1	30.1 ± 1.9	29.5 ± 1.6	28.9 ± 1.8
2	30.5 ± 1.8	30.1 ± 2.0	29.1 ± 1.6
3	31.1 ± 1.5	30.5 ± 1.7	29.0 ± 1.7
4	30.8 ± 1.6	30.2 ± 1.5	29.2 ± 1.7
5	30.2 ± 1.3	29.3 ± 1.7	29.2 ± 1.9
6	31.4 ± 1.7	30.2 ± 1.6	29.3 ± 2.2
7	30.9 ± 1.6	30.9 ± 1.9	29.9 ± 2.1
8	31.6 ± 2.0	31.2 ± 1.8	30.3 ± 2.1
9	31.3 ± 2.4	31.0 ± 2.1	31.1 ± 2.4

**Table 2:** Physiological parameters.

Group	Blood glucose (mmol/L)	Blood triglyceride (mmol/L)	Gastrocnemius muscle mass (mg)	Gastrocnemius muscle index
C	14.526 ± 1.53	2.494 ± 0.22	182.4 ± 10.9	0.011438 ± 0.000537
M	14.025 ± 1.56	2.505 ± 0.20	185.3 ± 7.5	0.011638 ± 0.000785
H	13.531 ± 1.57	2.385 ± 0.30	181.0 ± 8.4	0.011730 ± 0.001346

### Gene expression analysis

**Fig 1:**
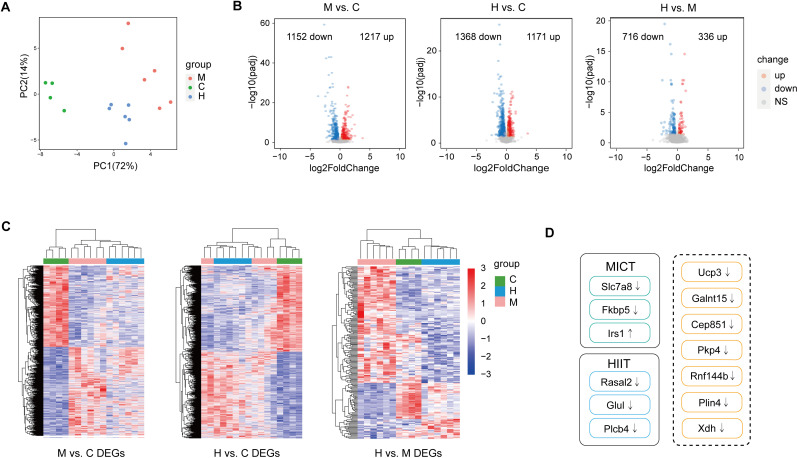
Gastrocnemius muscle transcriptome analysis. (A) PCA of total genes in C, M, H groups. (B) Volcano plots show DEGs in M vs. C, H vs. C and H vs. M. (C) Hierarchical clustering analysis of DEGs (M vs. C, H vs. C, H vs. M) in C, M, H groups. (D) The top 10 significant DEGs in M vs. C and H vs. C. Yellow indicates the common DEGs.

As shown in Fig 1A, the principal components analysis (PCA) indicated clear separation among the C, M, and H groups, suggesting significant differences among the three groups and high consistency within each group.

We applied a filtering criteria of adjusted *p* value < 0.05 and | log_2_foldchange | ≥ 0.3 for this study. In total, 2369 (1217 up-regulated, 1152 down-regulated), 2539 (1171 up-regulated, 1368 down-regulated) and 1052 (336 up-regulated, 716 down-regulated) DEGs were identified in M vs. C, H vs. C and H vs. M, respectively (Fig 1B, S2–S4 Tables). The expression of DEGs in the three groups for each comparison was presented using heatmap (Fig 1C).

The top 10 significant DEGs in M vs. C were *Ucp3*, *Galnt15*, *Slc7a8*, *Fkbp5*, *Rnf144b*, *Cep85l*, *Pkp4*, *Irs1*, *Xdh*, *Plin4* (Table 3). The top 10 significant DEGs in H vs. C were *Rnf144b*, *Galnt15*, *Plin4*, *Ucp3*, *Xdh*, *Rasal2*, *Cep85l*, *Pkp4*, *Glul*, *Plcb4* (Table 4). 7 out of 10 of the most significant DEGs in M vs. C were also detected in H vs. C (Fig 1D), suggesting a substantial overlap in the transcriptomic adaptation elicited by the two exercise modalities. The top 10 significant DEGs in H vs. M were *Apold1*, *Dusp10*, *Myod1*, *Ucp3*, *Fkbp5*, *Klf2*, *Zfp872*, *Plxna2*, *Ldlr*, *Foxo1* (Table 5).

**Table 3:** The top 10 significant DEGs in M vs. C.

Gene Symbol	Official Full Name	log2 (FC)	adjusted p value	Regulation
*Ucp3*	Uncoupling protein 3	−2.64243	1.22E-59	down
*Galnt15*	Polypeptide N-acetylgalactosaminyltransferase 15	−1.46987	2.05E-43	down
*Slc7a8*	Solute carrier family 7 member 8	−1.83976	2.05E-43	down
*Fkbp5*	FKBP prolyl isomerase 5	−2.33283	8.17E-43	down
*Rnf144b*	Ring finger protein 144B	−1.35914	1.59E-35	down
Cep85l	Centrosomal protein 85 like	−1.88382	1.75E-34	down
*Pkp4*	Plakophilin 4	−1.23892	4.37E-30	down
*Irs1*	Insulin receptor substrate 1	1.361908	1.34E-28	up
*Xdh*	Xanthine dehydrogenase	−1.20137	1.85E-28	down
*Plin4*	Perilipin 4	−1.6394	2.34E-28	down

**Table 4:** The top 10 significant DEGs in H vs. C.

Gene Symbol	Official Full Name	log2 (FC)	adjusted p value	Regulation
*Rnf144b*	Ring finger protein 144B	−1.19462	1.98E-26	down
*Galnt15*	Polypeptide N-acetylgalactosaminyltransferase 15	−1.069	4.18E-22	down
*Plin4*	Perilipin 4	−1.35854	1.61E-18	down
*Ucp3*	Uncoupling protein 3	−1.39597	1.48E-15	down
*Xdh*	Xanthine dehydrogenase	−0.90166	3.69E-15	down
*Rasal2*	RAS protein activator like 2	−1.0771	5.26E-15	down
*Cep85l*	Centrosomal protein 85 like	−1.2584	1.44E-14	down
*Pkp4*	Plakophilin 4	−0.84769	2.35E-13	down
*Glul*	Glutamate-ammonia ligase	−1.2254	1.69E-12	down
*Plcb4*	Phospholipase C beta 4	−0.62484	5.60E-12	down

**Table 5:** The top 10 significant DEGs in H vs. M.

Gene Symbol	Official Full Name	log2 (FC)	adjusted p value	Regulation
*Apold1*	Apolipoprotein L domain containing 1	−2.04152	2.55E-20	down
*Dusp10*	Dual specificity phosphatase 10	−1.11973	5.72E-17	down
*Myod1*	Myogenic differentiation 1	−0.94908	2.45E-15	down
*Ucp3*	Uncoupling protein 3	1.246461	2.46E-15	up
*Fkbp5*	FKBP prolyl isomerase 5	1.120498	5.37E-11	up
*Klf2*	KLF transcription factor 2	−1.02768	5.37E-11	down
*Zfp872*	Zinc finger protein 872	−2.42528	5.37E-11	down
*Plxna2*	Plexin A2	0.901478	5.42E-11	up
*Ldlr*	Low density lipoprotein receptor	−0.94415	2.18E-10	down
*Foxo1*	Forkhead box O1	0.972113	2.18E-10	down

### GO functional classification

To elucidate the biological functions of these DEGs, we performed gene ontology (GO) functional classification. Using a q value threshold of less than 0.01, we identified 466, 255 and 54 significantly enriched terms in M vs. C, H vs. C and H vs. M, respectively.

**Fig 2:**
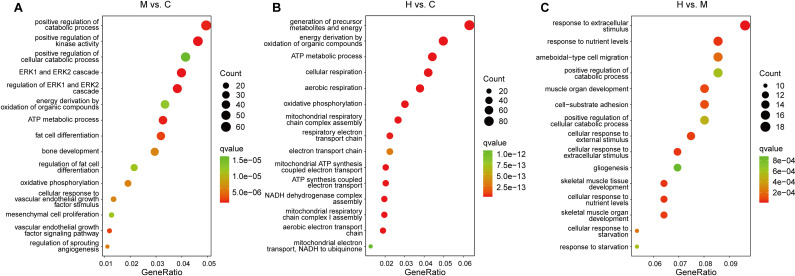
GO function classification (biological process) of DEGs. (A) M vs. C, (B) H vs. C, (C) H vs. M.

In M vs. C, the top 15 significantly enriched terms (biological process) included positive regulation of kinase activity, regulation of ERK1 and ERK2 cascade, ERK1 and ERK2 cascade, ATP metabolic process, positive regulation of catabolic process, fat cell differentiation, vascular endothelial growth factor signaling pathway, regulation of sprouting angiogenesis, oxidative phosphorylation, cellular response to vascular endothelial growth factor stimulus, bone development, mesenchymal cell proliferation, regulation of fat cell differentiation, energy derivation by oxidation of organic compounds, positive regulation of cellular catabolic process (Fig 2A, S5 Table).

In H vs. C, the top 15 significantly enriched terms (biological process) were aerobic respiration, generation of precursor metabolites and energy, cellular respiration, oxidative phosphorylation, energy derivation by oxidation of organic compounds, mitochondrial respiratory chain complex assembly, ATP metabolic process, NADH dehydrogenase complex assembly, mitochondrial respiratory chain complex I assembly, aerobic electron transport chain, mitochondrial ATP synthesis coupled electron transport, ATP synthesis coupled electron transport, respiratory electron transport chain, electron transport chain, mitochondrial electron transport NADH to ubiquinone (Fig 2B, S6 Table).

In H vs. M, the top 15 significantly enriched terms (biological process) included response to extracellular stimulus, response to nutrient levels, cellular response to extracellular stimulus, skeletal muscle tissue development, cellular response to nutrient levels, skeletal muscle organ development, cellular response to external stimulus, muscle organ development, cell-substrate adhesion, ameboidal-type cell migration, cellular response to starvation, positive regulation of cellular catabolic process, response to starvation, positive regulation of catabolic process, gliogenesis (Fig 2C, S7 Table).

### KEGG pathway analysis

To deepen our comprehension of these DEGs, we employed Kyoto Encyclopedia of Genes and Genomes (KEGG) pathway analysis to elucidate their roles in biological pathways. Using a q-value threshold of less than 0.05, we identified significant enrichment of 46, 45, and 4 pathways in M vs. C, H vs. C, and H vs. M, respectively.

**Fig 3:**
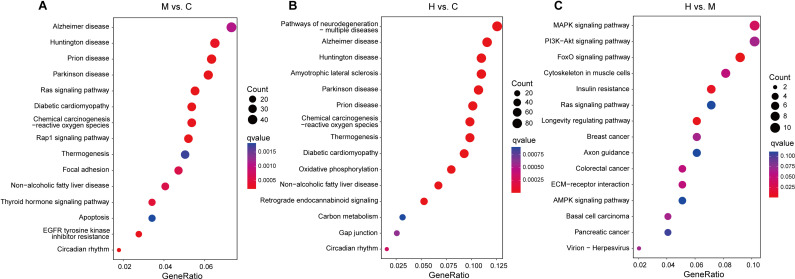
KEGG pathway analysis of DEGs. (A) M vs. C, (B) H vs. C, (C) H vs. M.

In M vs. C, the top 15 significantly enriched pathways were prion disease, diabetic cardiomyopathy, Parkinson disease, Rap1 signaling pathway, circadian rhythm, Ras signaling pathway, chemical carcinogenesis-reactive oxygen species, EGFR tyrosine kinase inhibitor resistance, Huntington disease, thyroid hormone signaling pathway, focal adhesion, non-alcoholic fatty liver disease, Alzheimer disease, thermogenesis, apoptosis (Fig 3A and S8 Table).

In H vs. C, the top 15 significantly enriched pathways included oxidative phosphorylation, chemical carcinogenesis-reactive oxygen species, Parkinson disease, thermogenesis, diabetic cardiomyopathy, Huntington disease, prion disease, Alzheimer disease, amyotrophic lateral sclerosis, non-alcoholic fatty liver disease, pathways of neurodegeneration, retrograde endocannabinoid signaling, circadian rhythm, gap junction, carbon metabolism (Fig 3B and S9 Table).

In H vs. M, the significantly enriched pathways were FoxO signaling pathway, longevity regulating pathway, insulin resistance, MAPK signaling pathway (Fig 3C and S10 Table).

### Myokines and muscle fiber markers

**Fig 4:**
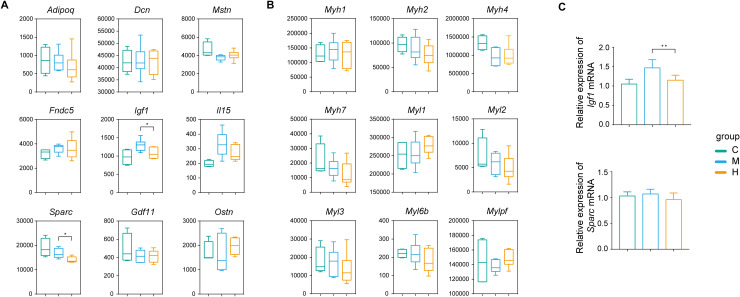
The expression levels of myokines and muscle fiber markers. (A) The expression levels (RNA-seq counts) of myokines and (B) muscle fiber markers in C, M, H groups. (C) Expression of *Igf-1* and *Sparce* mRNA in C, M, H, groups’ gastrocnemius muscle (each group n = 6); results are shown as relative expression normalized to that of *Gapdh* mRNA. *p < 0.05, **p < 0.01.

Myokines, produced by skeletal muscle cells, represent a class of cytokines or peptides [15]. Research has suggested that myokines play pivotal roles in modulating inflammation, lipid metabolism, energy metabolism, and overall health [16,17]. Therefore, we analyzed the expression levels of various myokines, including adiponectin (*Adipoq*), decorin (*Dcn*), myostatin (*Mstn*), fibronectin type III domain-containing protein 5 (*Fndc5*), insulin-like growth factor 1 (*Igf1*), interleukin 15 (*Il15*), secreted protein acidic and rich in cysteine (*Sparc*), growth differentiation factor 11 (*Gdf11*), and osteocrin (*Ostn*). Among these, the expression levels of *Igf-1* and *Sparc* were significantly different between the two exercise modalities (Fig 4A). Subsequently, we analyzed the expression levels of various markers of muscle fiber: *Myh1* (IIX), *Myh2* (IIA), *Myh4* (IIB), *Myh7* (I), *Myl1* (II), *Myl2* (I), *Myl3* (I), *Myl6b*(I), and *Mylpf* (II). There was no notable variance between the two exercise modalities (Fig 4B). Furthermore, quantitative RT-PCR was performed to validate the RNA-seq results. Consistently, *Igf-1* mRNA level was significantly higher in the M group compared to the H group, while no significant difference was observed in *Sparc* mRNA expression (Fig 4C).

## Discussion

Skeletal muscle, as the primary organ responsible for movement, plays a critical role during physical activity. Its adaptive changes in response to exercise not only manifest as alterations in muscle structure and function but also entail genetic regulation and adaptation [18,19]. Understanding how skeletal muscle adapts at the genetic level to different types of exercise is crucial for optimizing training strategies and improving health outcomes.

In this study, we investigated the effects of 8 weeks of treadmill-based MICT and HIIT on the transcriptome of murine skeletal muscle. No significant differences in body weight, glucose and triglycerides were observed between the C group and exercise groups. This was likely attributed to the use of 14-week-old C57BL/6J mice. At this age, mice have achieved stable body weight and metabolic homeostasis, making them a suitable model for studying skeletal muscle adaptations to exercise while minimizing potential confounding effects related to development.

Applying the filtering criteria of adjusted *p* value < 0.05 and | log_2_foldchange | ≥ 0.3, 1052 DEGs were identified in H vs. M. Among the top 10 significant DEGs, *Foxo1* (mean counts: C, 4616; M, 1413; H, 2772) is of particular interest. FoxO1 is a transcription factor that regulates the expression of genes involved in muscle protein degradation and cellular processes associated with muscle wasting, such as autophagy and the ubiquitin-proteasome system [20]. Target genes of FoxO1 in muscle atrophy include those encoding for proteins involved in muscle protein breakdown, such as *Murf1* (muscle-specific RING finger protein 1) and *Atrogin-1*, as well as genes related to autophagy and oxidative stress response [21,22]. Increased FoxO1 activity is a hallmark of muscle atrophy, contributing to the breakdown of muscle proteins and leading to a loss of muscle mass and function [23]. Activated AKT phosphorylates FoxO1 directly, leading to its exclusion from the nucleus, thus inhibiting FoxO1’s transcriptional activity. Other significant DEG related to muscular physiology is *Myod1* (mean counts: C, 529; M, 1390; H, 720). MyoD is a member of the myogenic regulatory factors (MRFs) family, which plays a critical role in muscle development and regeneration [24]. As a transcription factor, MyoD regulates the expression of genes involved in muscle differentiation, including those encoding structural proteins essential for muscle formation. It acts as a master regulator by activating the muscle-specific gene program and promoting the conversion of undifferentiated cells into muscle cells (myoblasts) [25]. MyoD is particularly important during embryonic development for the initial specification of skeletal muscle lineage and in adult skeletal muscle for the repair and regeneration of damaged muscle tissue [26].

Beyond the gene level, GO functional classification revealed that the majority of DEGs in H vs. M were associated with extracellular stimulus, nutrient levels, and skeletal muscle development. The significantly enriched pathways in H vs. M were FoxO signaling pathway, longevity regulating pathway, insulin resistance, MAPK signaling pathway. Interestingly, these pathways are closely associated with PI3K-AKT pathway and its upstream regulator, IGF-1 [27]. IGF-1, a protein hormone structurally akin to insulin, profoundly contributes to muscle growth, repair, and regeneration by acting as a potent stimulator of muscle protein synthesis [28,29]. It interacts specifically with the IGF receptor situated on the cell surface, initiating a cascade of events. Upon ligand binding, the receptor undergoes a conformational change in its β subunit, activating its tyrosine kinase activity. Consequently, the activated receptor phosphorylates various substrates, including insulin receptor substrates (IRSs) and Src homology collagen (SHC) proteins. These substrates, in turn, initiate two primary intracellular signaling pathways: the PI3K/AKT pathway and the MAPK pathway.

**Fig 5:**
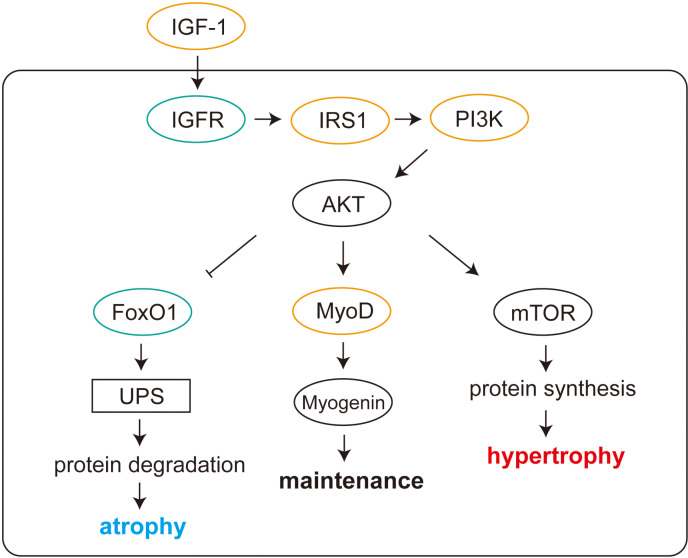
IGF1-PI3K-AKT pathway. orange/green indicates significantly higher/lower expression levels in M group than that in H group. UPS, ubiquitin-proteasome system.

While IGF-1 is primarily synthesized in the liver and released into the bloodstream, muscle tissue also produces and secretes IGF-1 [30]. In muscle tissue, the synthesis of IGF-1 is regulated by stimulation (e.g., exercise [31]). We found that the expression level of *Igf-1* in M group was significantly higher than that in H group (Fig 4A,C). As depicted in Fig 5, several key components of IGF1-PI3K-AKT pathway were also expressed at higher levels in M group, indicating a potentially heightened activation of IGF1-PI3K-AKT pathway induced by MICT. Since it has been reported that downregulation of IGF1-PI3K-AKT signaling pathway and increased FoxO activity were often associated with muscle atrophy in diseased states or in elderly individuals [32–34], our findings suggest that MICT might offer superior benefits compared to HIIT in ameliorating muscle atrophy under such conditions.

Limitations: One limitation of this study is the relatively small sample size (n = 12 per group), which may reduce the statistical power to detect subtle differences and increase the potential impact of individual variability in exercise response. Future studies with larger sample sizes are necessary to validate these findings and ensure greater generalizability. Additionally, the use of a murine model, while informative, may not fully replicate the adaptive responses in human skeletal muscle due to species-specific differences. Validation of these findings in human models or across different age groups would further enhance the applicability of the results.

The intervention lasted only 8 weeks, which, while sufficient to observe short-term transcriptomic adaptations, may not capture the full extent of long-term effects, such as sustained improvements in muscle function or metabolic health. Future studies with extended training durations are needed to better elucidate these longer-term adaptations.

Furthermore, the exercise volume in this study was controlled by keeping the running distance constant across groups. However, exercise intensity, duration, and distance are all critical variables that can influence adaptive outcomes. By focusing on a fixed distance, this study may have overlooked the broader spectrum of responses that could result from variations in these parameters, especially differences in energy expenditure or recovery dynamics between the exercise modalities.

Lastly, this study relied exclusively on transcriptomic analysis, which provides valuable insights into gene expression changes but does not directly capture post-transcriptional modifications, protein abundance, or metabolite interactions. Integrating proteomic and metabolomic analyses in future studies could offer a more comprehensive understanding of the molecular mechanisms underlying skeletal muscle adaptations to different exercise modalities.

## Conclusions

This study sheds light on the transcriptomic landscape of skeletal muscle in response to MICT and HIIT, highlighting distinct adaptations in the FoxO, MAPK, and PI3K-AKT signaling pathways. These differences might be attributed to variations in *Igf-1* expression elicited by the two exercise modalities.

## Supporting information

S1 Table VO_2_max test. (DOCX)

S2 Table Deseq2 result M vs. C. (CSV)

S3 Table Deseq2 result H vs. C. (CSV)

S4 Table Deseq2 result H vs. M. (CSV)

S5 Table GO result M vs. C. (CSV)

S6 Table GO result H vs. C. (CSV)

S7 Table GO result H vs. M. (CSV)

S8 Table KEGG result M vs. C. (CSV)

S9 Table KEGG result H vs. C. (CSV)

S10 Table KEGG result H vs. M. (CSV)
