# Supplementary material for: Transcriptomic adaptation of skeletal muscle in response to MICT and HIIT exercise modalities
Source: PLoS One. 2025 Feb 25;20(2):e0318782. doi: 10.1371/journal.pone.0318782 (PMC11856427; doi:10.1371/journal.pone.0318782)
Supplement: S1 Table — (DOCX) [file pone.0318782.s001.docx]

**S1 Table 1. VO_2_max test**

| Training week | 1-2 | 3-4 | 5-6 | 7-8 |
| --- | --- | --- | --- | --- |
| VO_2_max（mL/h/kg） | 7112±159 | 7235±187 | 7367±138 | 7355±166 |
| VO_2_max（m/min） | 27 | 30 | 33 | 33 |
| 60%VO_2_max（m/min） | 16 | 18 | 20 | 20 |
| 90%VO_2_max（m/min） | 24 | 27 | 30 | 30 |

The VO_2_max test began with an initial speed set at 3 m/min and a 0° incline, lasting for 5 minutes as a warm-up phase. Afterward, the speed was increased by 3 m/min every minute. During the incremental load exercise test, the criteria for reaching VO_2_max were met when any two of the following conditions were satisfied:

1) VO_2_ no longer increased with the increasing exercise load, forming a plateau or showing a change of no more than 5%;

2) the respiratory exchange ratio exceeded 1.0;

3) the mice reached exhaustion, demonstrated by the inability to keep up with the treadmill speed, contact of the abdomen with the treadmill surface, weakness in hindlimb push-off, and lack of response to electrical stimulation or manual encouragement.
